# Longitudinal Lipidomic Profile of Subclinical Peripheral Artery Disease in American Indians: The Strong Heart Family Study

**DOI:** 10.5888/pcd22.240220

**Published:** 2025-05-08

**Authors:** Mingjing Chen, Guanhong Miao, Mary J. Roman, Richard B. Devereux, Richard R. Fabsitz, Ying Zhang, Jason G. Umans, Shelley A. Cole, Oliver Fiehn, Jinying Zhao

**Affiliations:** 1 Department of Epidemiology, College of Public Health & Health Professions, University of Florida, Gainesville; 2 Division of Cardiology, Weill Cornell Medical College, New York, New York; 3 Missouri Breaks Industries Research, Inc, Eagle Butte, South Dakota; 4 Department of Biostatistics and Epidemiology, University of Oklahoma Health Sciences Center, Oklahoma City; 5 MedStar Health Research Institute, Hyattsville, Maryland; 6 Georgetown-Howard Universities Center for Clinical and Translational Science, Washington, District of Columbia; 7 Texas Biomedical Research Institute, San Antonio, Texas; 8 West Coast Metabolomics Center, University of California, Davis

## Abstract

**Introduction:**

Peripheral artery disease (PAD) and dyslipidemia are both independent predictors of cardiovascular disease, but the association between individual lipid species and subclinical PAD, assessed by ankle-brachial index (ABI), is lacking in large-scale longitudinal studies.

**Methods:**

We used liquid chromatography-mass spectrometry to repeatedly measure 1,542 lipid species from 1,886 American Indian adults attending 2 clinical examinations (mean ~5 years apart) in the Strong Heart Family Study. We used generalized estimating equation models to identify baseline lipid species associated with change in ABI and the Cox frailty regression to examine whether lipids associated with change in ABI were also associated with incident coronary heart disease (CHD). We also examined the longitudinal association between change in lipid species and change in ABI and the cross-sectional association of individual lipids with ABI. All models were adjusted for age, sex, body mass index, smoking, alcohol use, hypertension, estimated glomerular filtration rate, diabetes, and lipid-lowering medication.

**Results:**

Baseline levels of 120 lipid species, including glycerophospholipids, glycerolipids, fatty acids, and sphingomyelins, were associated with change in ABI. Among these, higher baseline levels of 3 known lipids (phosphatidylinositol[16:0/20:4], triacylglycerol[48:2], triacylglycerol[55:1]) were associated with a lower risk of CHD (hazard ratios [95% CIs] ranged from 0.67 [0.46–0.99] to 0.76 [0.58–0.99]), while cholesterol was associated with a higher risk of CHD (hazard ratio [95% CI] = 1.37 [1.00–1.87]). Longitudinal changes in 32 lipids were significantly associated with change in ABI during 5-year follow-up. Plasma levels of glycerophospholipids, triacylglycerols, and glycosylceramides were significantly associated with ABI in the cross-sectional analysis.

**Conclusion:**

Altered plasma lipidome is significantly associated with subclinical PAD in American Indians beyond traditional risk factors. If validated, the identified lipid species may serve as novel biomarkers for PAD in this high-risk but understudied population.

**Summary**

**What is already known on this topic?**

Peripheral artery disease (PAD) and dyslipidemia are both independent predictors of cardiovascular disease (CVD). Lipidomics can identify and quantify individual lipid species associated with subclinical PAD.

**What is added by this report?**

This is the first longitudinal lipidomic study of subclinical PAD in a large community‐based cohort of American Indians. Altered baseline levels of multiple individual lipid species and their changes were associated with subclinical PAD, with some lipids also associated with coronary heart disease risk beyond traditional risk factors.

**What are the implications for public health practice?**

Given the high prevalence of CVD risk factors among American Indians, early screening for PAD at younger ages is essential.

## Introduction

Lower extremity peripheral artery disease (PAD) is characterized by a partial or complete obstruction of lower limb arteries by atherosclerotic blockages. PAD affects more than 200 million adults globally and poses a substantial burden to public health (1). Patients with atherosclerotic PAD are at increased risk of myocardial infarction, stroke, and death. American Indians have a higher prevalence than non-Hispanic White people of inpatient PAD and chronic limb-threatening ischemia (2). Dyslipidemia, defined as high levels of total or low-density lipoprotein (LDL) cholesterol or low levels of high-density lipoprotein (HDL) cholesterol, is associated with PAD (3). Traditional lipid panels measure only bulk lipoproteins and fail to reflect the diverse molecular lipid species in a blood sample. A comprehensive profiling of all individual lipid species in our blood (ie, blood lipidome) is required to identify novel biomarkers and enhance our understanding of the mechanism through which dyslipidemia may contribute to atherosclerotic PAD.

Lipidomics is an emerging high-throughput biochemical technique that can identify and quantify hundreds to thousands of molecular lipid species in biofluids or tissues. Epidemiologic studies have used lipidomics to describe associations of altered lipid species, such as ceramides (CERs), cholesterol, phospholipids, and fatty acids (FAs) with PAD in human populations (4,5). However, these studies were largely cross-sectional, had small sample sizes, and/or had low coverage of the blood lipidome. To our knowledge, no large-scale longitudinal lipidomic study has investigated the association between longitudinal change in blood lipidome and change in ankle-brachial index (ABI), a sensitive and cost-effective tool for PAD screening, in any racial or ethnic group. The normal range for ABI is generally 0.9 to 1.4; an ABI <0.9 typically indicates occlusive PAD related to atherosclerosis, while an ABI >1.4 reflects noncompressible vessels, which may still suggest underlying occlusive disease (6). Although the prevalence of ABI >1.4 among adults aged 40 years or older (mean age, 56.9 y) is relatively low (approximately 1.4%) in the general US population (7), data from the Strong Heart Study cohort (8) showed a higher prevalence of ABI >1.4 (9.2%) among American Indians of similar age (mean age, 57.1 y). The higher prevalence among American Indians may be largely attributed to the higher rates of obesity and diabetes.

The objective of our study was to investigate 1) whether changes in individual lipid species are associated with change in ABI over an average of 5-year follow-up among American Indians, independent of baseline lipids and traditional risk factors; and 2) whether ABI-related lipid species are associated with incident CHD during an average of 18-year follow-up over traditional risk factors. We also analyzed the cross-sectional association of each lipid species with ABI at baseline and 5-year follow-up.

## Methods

We conducted the first large-scale longitudinal lipidomic profiling of 3,645 fasting plasma samples from 1,886 unique American Indians attending 2 clinical examinations (1,886 at baseline; 1,759 at follow-up) approximately 5 years apart on average in the Strong Heart Family Study. By including participants with an ABI <0.9 or an ABI >1.4, we aimed to capture a broad spectrum of lipid changes associated with cardiovascular health, rather than focusing only on people with ABI values within the normal range.

### Study population

The Strong Heart Family Study (2001–ongoing) is a family-based prospective study designed to identify genetic, metabolic, and behavioral factors for cardiovascular disease (CVD) and CVD risk factors among American Indians (9). Briefly, 2,786 tribal members (aged ≥14 y) residing in Arizona, North Dakota, South Dakota, and Oklahoma were recruited and examined at baseline (2001–2003) and re-examined after 5-year follow-up (2006–2009). Details of the study design, laboratory protocols, and phenotype collection are available elsewhere (9). Participants were interviewed and had a physical examination at each visit, during which fasting blood samples were collected for laboratory tests. Laboratory methods were described previously (9). We included in analysis 1,886 individuals (62.2% females; mean age, 40.1 y) who were free of overt CVD at baseline and had complete clinical and lipidomic data. All study participants provided informed consent, and protocols were approved by the institutional review boards of participating institutions and the American Indian tribes.

### Measurement of ABI

At baseline and at 5-year follow-up, blood pressure was measured in the right arm and bilateral ankles (both left and right posterior tibial artery) by using a handheld Doppler (Imex Medical Limited) while the participant was in a supine position. Each measurement was taken twice in immediate succession, and the average of the 2 readings was used. If no pulse was detected either by palpation or Doppler, a second examiner was asked to confirm the absence and ankle blood pressure was obtained from the dorsalis pedis artery. The ABI for each leg was then calculated by dividing the average systolic blood pressure in the ankle (posterior tibial or dorsalis pedis) by the average systolic blood pressure in the right arm (brachial artery). The worse of the 2 ABI values (ie, the lower value for ABI <0.9 or the higher value for ABI >1.4) was used to define ABI for each person. Change in ABI was calculated as the difference in ABI between baseline and 5-year follow-up. Symptoms indicative of PAD, such as intermittent claudication, were evaluated at both baseline and follow-up by using the Rose Angina Questionnaire (10), which asks about symptoms such as leg pain during walking or resting and other signs of vascular insufficiency. PAD was defined as participants who had an ABI of either <0.9 or >1.4 in at least 1 leg during the clinical examination (6). Incident PAD was defined as not having PAD at baseline but having PAD at 5-year follow-up.

### Assessment of clinical covariates

Information on demographic characteristics (age, sex), lifestyle (smoking, alcohol use, physical activity), medical history, and use of prescription medications was collected via standard questionnaires (11). Anthropometric measures (height, weight, waist circumference) and fasting blood samples were collected at each visit. Smoking status was categorized as current, former, or never smokers, and alcohol use as current versus noncurrent drinkers. Hypertension was defined as blood pressure ≥140/90 mm Hg or use of antihypertensive medication, and type 2 diabetes as fasting glucose ≥126 mg/dL or use of hypoglycemic medication. CVD events included fatal and nonfatal myocardial infarction, CHD, sudden cardiac death, heart failure, and stroke. Use of lipid-lowering and antihypertensive medication was recorded at each visit.

### Ascertainment of incident coronary heart disease (CHD)

Details on the ascertainment of incident CHD are available elsewhere (12). In brief, CHD included definite CHD (fatal or nonfatal), definite myocardial infarction (fatal or nonfatal), and sudden death due to CHD. CHD events were ascertained by annual review of hospitalizations, death records, and self-reports (with subsequent medical record verification) during follow-up visits. Time to event was recorded based on the date of baseline examination (2001–2003) to either the date of the first CHD event or the last follow-up (December 31, 2020). For participants who experienced more than 1 CHD event during an average 18-year follow-up period, we used the earliest event date in our analysis.

### Lipidomic data acquisition, preprocessing, and quality control

Methods for blood sample collection, lipidomic data acquisition, processing, and normalization are described elsewhere (13). Briefly, relative abundance of molecular lipid species in fasting plasma samples at 2 time points (~5 years apart) was quantified by untargeted liquid chromatography–mass spectrometry. After preprocessing and quality control, we obtained 1,542 lipids (518 known) in 3,950 samples (1,970 at baseline; 1,980 at 5-year follow-up). After further excluding outlier samples (n = 2 at baseline; n = 3 at follow-up) and people with prevalent CVD (n = 12 at baseline; n = 87 at follow-up) or missing covariates (n = 70 at baseline; n = 131 at follow-up), we included 1,886 participants (1,886 at baseline; 1,759 at 5-year follow-up) with complete clinical and lipidomic data. We observed no clear batches in our lipidomic data.

### Statistical analysis

Continuous variables, including lipid levels, were standardized to zero mean and unit variance. Multiple testing was controlled by false discovery rate by using the Storey *Q*-value method (14); *Q* <.05 was used to determine significance.

#### Prospective association analysis

To identify baseline plasma lipids that can predict change in ABI (ie, the difference in ABI between baseline and 5-year follow-up), we constructed a generalized estimating equation (GEE) model, which accounted for the relatedness among family members. In this model, the baseline level of each lipid was the predictor, and change in ABI was the outcome, adjusting for age, sex, body mass index (BMI), smoking status (current smoker vs ever smoker vs nonsmoker), alcohol use (yes/no), hypertension (yes/no), diabetes (yes/no), estimated glomerular filtration rate (eGFR), use of lipid-lowering medication (yes/no) at baseline, and baseline ABI. We excluded participants with prevalent PAD at baseline from this analysis.

To assess whether the identified lipids improved the prediction of PAD risk beyond known clinical factors, we used data from 2 study centers (North and South Dakota and Arizona) as the training set (n = 995; 32 cases), and 1 center (Oklahoma) as the testing set (n = 788; 65 cases). We then compared a base model including traditional risk factors only (age, sex, BMI, smoking status, alcohol use, hypertension, diabetes, eGFR, and use of lipid-lowering medication) with a model that included both traditional risk factors and the lipids associated with change in ABI. We assessed the incremental predictive value of lipids over known risk factors by area under the receiver operating characteristic curve (AUROC) (15).

To further examine whether plasma lipids associated with change in ABI were also associated with incident CHD during an average of 18-year follow-up, we constructed a frailty Cox proportional hazards model. In this model, the baseline level of each ABI-associated lipid was the predictor, and the time to incident CHD event was the outcome, adjusting for the same covariates as described above, plus LDL cholesterol, HDL cholesterol, and physical activity at baseline. The frailty term was used to account for the relatedness among family members.

#### Repeated measurement analysis

For the 1,459 participants free of prevalent CVD and PAD at baseline and 5-year follow‐up, we constructed GEE models to examine the longitudinal association between changes in lipid species (difference in the relative abundance of each lipid) and change in ABI between 5-year follow-up and baseline. In the model, change in ABI was the outcome, and change in the relative abundance of a lipid was the predictor. The model adjusted for age, sex, BMI, smoking status (current smoker vs ever smoker vs nonsmoker), alcohol use (yes/no), hypertension (yes/no), eGFR, diabetes (yes/no), and use of lipid-lowering medication (yes/no) at baseline, as well as changes in continuous variables (ie, age, BMI, eGFR) and baseline lipid. The associations between changes in lipids and change in cardiometabolic factors, including BMI, systolic and diastolic blood pressure, eGFR, fasting blood plasma glucose, insulin, and insulin resistance, were similarly examined.

#### Cross-sectional association analysis

To identify lipids that are cross-sectionally associated with ABI at each point (baseline or 5-year follow-up), we constructed GEE models in which ABI was the outcome and the plasma level of each lipid was the predictor, adjusting for age, sex, BMI, smoking status (current smoker vs ever smoker vs nonsmoker), alcohol use (yes/no), hypertension (yes/no), eGFR, diabetes (yes/no), and lipid-lowering medication (yes/no). This analysis was conducted separately by using data collected at baseline or 5-year follow-up. Results from both points were then combined by fixed-effects meta-analysis.

#### Sensitivity analysis

To evaluate the robustness of our results, we conducted the following sensitivity analyses. First, to examine the potential effect of bulk lipids (ie, HDL cholesterol, triglycerides) and physical activity on our results for change in ABI or ABI at each point, we additionally adjusted for these variables in the models. Second, to examine whether sex modulated the association between lipid species and ABI or change in ABI, we further included an interaction term (lipid × sex) in the model. Third, to examine whether the inclusion of symptomatic participants or the potential effect of PAD symptoms affected our results, we performed additional analyses. First, we excluded participants who reported symptoms indicative of PAD from the models. We excluded 423 symptomatic participants from the prospective association analysis, 338 from the repeated measurement analysis, and 460 from the cross-sectional analysis. Second, we adjusted for PAD symptoms in the models.

## Results

The mean age of participants was 40.1 years at baseline and 44.9 years at follow-up (Table). The median ABI was 1.1 at baseline and 1.2 at follow-up, respectively. Most participants had an ABI within the normal range (0.9 ≤ ABI ≤ 1.4) at both baseline (1,828 of 1,886; 96.9%) and follow-up (1,652 of 1,759; 93.9%). Among the 1,828 participants free of PAD at baseline, 97 participants (5.3%) developed incident PAD during an average 5-year follow-up.

**Table:** Characteristics of Study Participants in the Strong Heart Family Study at Baseline (2001–2003) and 5-Year Follow-Up (2006–2009)^a^

Characteristics	Baseline (n = 1,886)	5-Year follow-up (n = 1,759)
Age, mean (SD), y	40.1 (13.9)	44.9 (13.4)
Female, no. (%)	1,175 (62.2)	1,103 (62.7)
Body mass index,^b^ mean (SD)	31.8 (7.5)	32.7 (7.7)
Current smoking, no. (%)	757 (40.1)	670 (38.3)
Current drinking, no. (%)	1,182 (62.6)	1,032 (58.9)
Type 2 diabetes, no. (%)	338 (17.9)	412 (23.4)
Systolic blood pressure, mean (SD), mm Hg	122.3 (15.3)	122.6 (16.3)
Diastolic blood pressure, mean (SD), mm Hg	77.3 (10.6)	74.9 (11.1)
eGFR, mean (SD), mL/min/1.73m^2^	114.9 (17.5)	108.6 (19.9)
High-density lipoprotein cholesterol, median (IQR), mg/dL	49.0 (42.0–59.0)	48.0 (40.0–58.3)
Low-density lipoprotein cholesterol, mean (SD), mg/dL	101.7 (29.9)	106.2 (30.7)
Triglycerides, median (IQR), mg/dL	138.0 (99.0–195.0)	133.0 (96.5–188.0)
Total cholesterol, median (IQR), mg/dL	182.0 (162.0–205.0)	186.3 (161.0–210.0)
Ankle brachial index, median (IQR)^c^	1.1 (1.1–1.2)	1.2 (1.1–1.3)
Physical activity, median (IQR), steps per day	5,147.5 (3,325.2–7,516.9)	5,841.0 (3,838.0–8,087.0)
Lipid-lowering medication, no. (%)	61 (3.2)	172 (9.8)
Antihypertensive medication, no. (%)	199 (10.6)	353 (20.1)

Abbreviations: eGFR, estimated glomerular filtration rate.

a Values are as mean (SD) for normally distributed data or median (IQR) for nonnormally distributed data. Categorical variables are expressed as number (percentage).

b Measured as weight in kilograms divided by height in meters squared.

c Ankle brachial index is the ratio of the systolic blood pressure in the ankle to the systolic blood pressure in the arm; the normal range is 0.9 to 1.4.

### Baseline lipids predict change in ABI beyond known clinical factors

**Figure 1:**
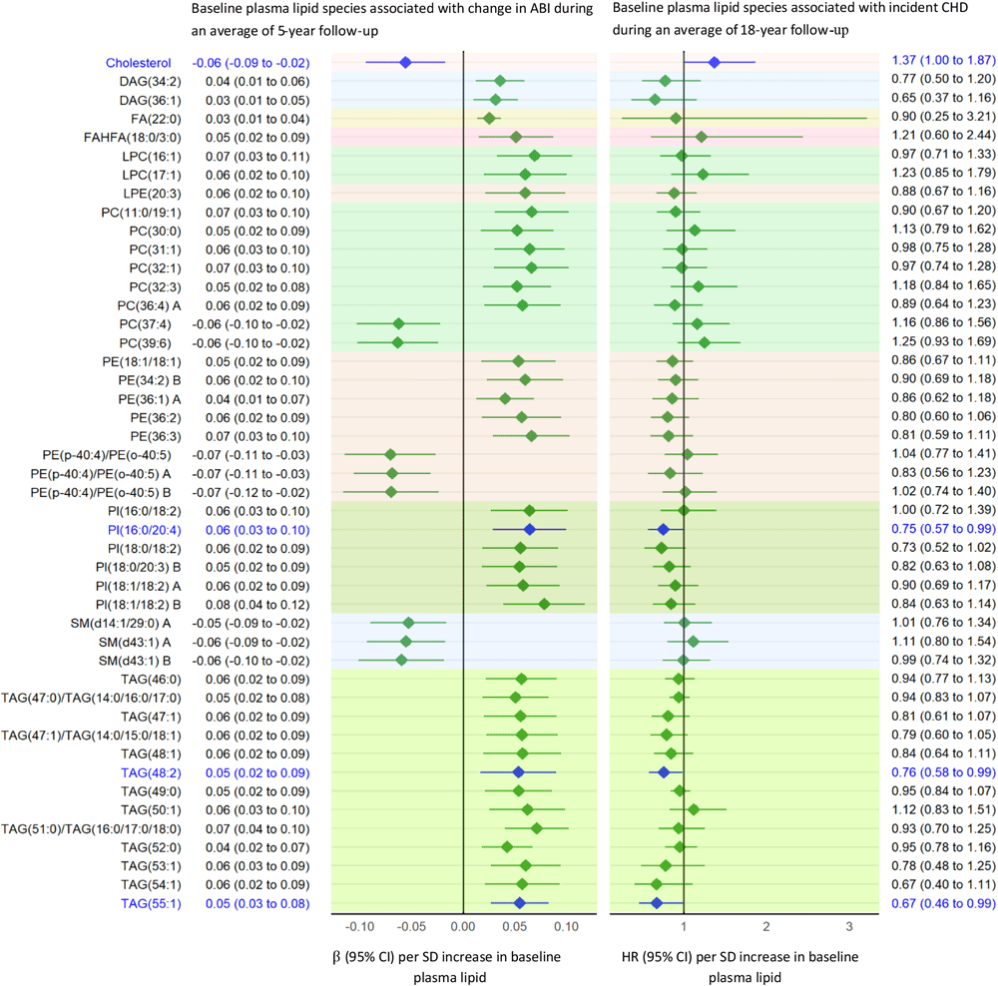
Baseline plasma lipid species associated with change in ABI (*Q* < .05). Lipids significantly associated with incident CHD are highlighted in blue. “A” or “B” in name of lipids indicates isomers. Abbreviations: ABI, ankle-brachial index; CHD, coronary heart disease; DAG, diacylglycerol; eGFR, estimate glomerular filtration rate; HR, hazard ratio; FA, fatty acid; FAHFA, fatty acid ester of hydroxy fatty acid; LPC, lysophosphatidylcholine; LPE, lysophosphatidylethanolamine; PC, phosphatidylcholine; PE, phosphatidylethanolamine; PI, phosphatidylinositol; SM, sphingomyelin; TAG, triacylglycerol.

We identified 358 lipids (143 known) significantly associated with change in ABI at *P* < .05. After correction for multiple testing, 120 lipids (46 known: 13 triacylglycerols [TAGs], 10 phosphatidylcholines [PCs], 9 phosphatidylethanolamines [PEs], 6 phosphatidylinositols [PIs], 3 sphingomyelins [SMs], 2 diacylglycerols [DAGs], fatty acid [FA{22:0}], fatty acid ester of hydroxy fatty acid [FAHFA {18:0/3:0}], and cholesterol, were significantly associated with change in ABI at *Q* <.05. Of the 46 known lipids, higher baseline levels of the following 37 lipids — 13 TAGs, 8 PCs, 6 PIs, 6 PEs, 2 DAGs, FA(22:0), and FAHFA(18:0/3:0) — were positively associated with change in ABI (regression coefficient [β] = 0.03–0.08). In contrast, higher baseline levels of 9 lipids (3 PEs, 3 SMs, 2 PCs, and cholesterol) were inversely associated with change in ABI (β, −0.05 to −0.07) (Figure 1).

**Figure 2:**
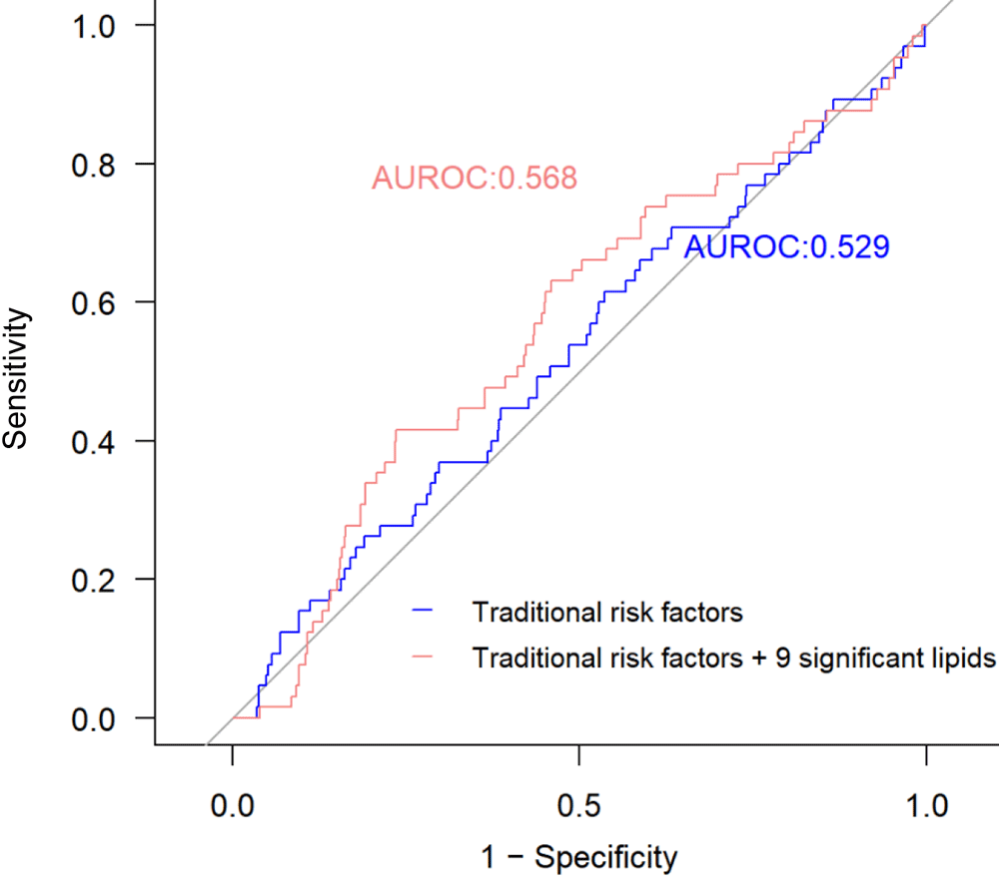
Incremental value of the identified plasma lipids associated with change in ABI for PAD risk prediction. Data used from 2 study centers (North and South Dakota and Arizona) as training set (n = 995, 32 cases), used for model training, and 1 center (Oklahoma) as the testing set (n = 788, 65 cases), used to test classification performance. Model 1 (blue line): traditional risk factors only, including age, sex, body mass index, smoking status, alcohol use, hypertension, diabetes, eGFR, and lipid-lowering medication use at baseline. Model 2 (red line): clinical factors plus 9 lipids significantly associated with change in ABI. Compared with Model 1, additional inclusion of plasma lipids (Model 2) significantly increased risk prediction for PAD; *P* value for increase in AUROC = .04. Abbreviations: ABI, ankle-brachial index; AUROC, area under the receiver operating characteristic curve; eGFR, estimated glomerular filtration rate, PAD, peripheral artery disease.

Addition of the top 9 of 46 lipids associated with change in ABI, namely FA(22:0), FAHFA(18:0/3:0), LPC(16:1), PI(16:0/20:4), PI(18:1/18:2) B, PI(18:0/18:2), PI(18:0/20:3) B, SM(d43:1) B, and LPE(20:3), significantly improved risk prediction for PAD over clinical factors (AUROC increased from 0.529 to 0.568, *P* = .04) (Figure 2).

### ABI-related lipids associated with incident CHD

Ninety study participants developed incident CHD during an average of 18 years of follow-up. Of the 46 known lipids whose baseline levels are associated with change in ABI during 5-year follow-up, baseline levels of 4 lipids were also significantly associated with risk of CHD at *P* <.05, after adjusting for age, sex, BMI, smoking, alcohol use, diabetes, hypertension, eGFR, LDL cholesterol, HDL cholesterol, physical activity, and use of lipid-lowering medication at baseline. Specifically, higher baseline levels of 3 known lipids, TAG(48:2), TAG(55:1), and PI(16:0/20:4), were associated with a decreased risk of CHD (hazard ratio [95% CI] ranged from 0.67 [0.46–0.99] to 0.76 [0.58–0.99]), while a higher baseline level of cholesterol was associated with an increased risk of CHD (hazard ratio [95% CI] = 1.37 [1.00–1.87]) during an average of 18-years follow-up.

### Longitudinal changes in lipid species associated with change in ABI during 5-year follow-up

**Figure 3:**
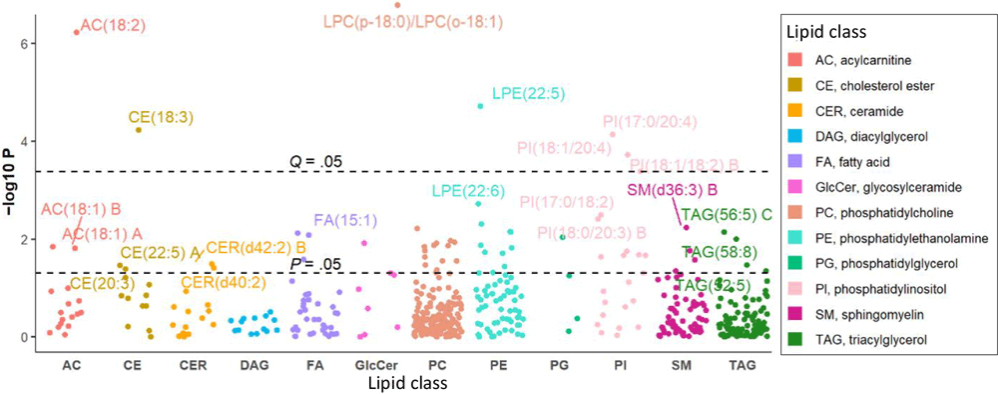
Manhattan plot displaying the longitudinal associations between change in plasma lipids and change in ABI during an average of 5-years follow-up. The dashed lines represent significance level at *P* = .05 and *Q* = .05. Abbreviation: ABI, ankle-brachial index.

After adjusting for clinical covariates, baseline ABI, and baseline lipids, longitudinal changes in 188 lipids (61 known) were significantly associated with change in ABI at *P* <.05. After correction for multiple testing, changes in 32 lipids (7 known) remained significant at *Q* <.05. Of the 7 known lipids, 6 lipids, including 3 PIs, AC(18:2), CE(18:3), and LPE(22:5), were positively associated with change in ABI at *Q* <.05, whereas change in LPC(p-18:0)/LPC(o-18:1) was inversely associated (Figure 3). Among lipid species associated with change in ABI, changes in most were also associated with changes in cardiovascular risk factors.

### Lipids cross-sectionally associated with ABI

At baseline, 310 lipids (128 known) were associated with ABI at *P* <.05. Of the 128 known lipids, 123 lipids, including 37 TAGs, 24 PCs, 17 PEs, 14 PIs, 10 DAGs, 8 acylcarnitine (ACs), 6 FAs, 2 CERs, SM(d32:2) A, GlcCer(d14:1(4E)/20:0(2OH)) and CE(22:5) B were inversely associated with ABI, whereas 5 lipids, including 2 PCs (LPC[20:0], LPC[o-16:0]), 2 PEs (PE[p-18:0/22:4]/PE[o-18:1/22:4], PE[p-40:4]/PE[o-40:5] A), and PS(18:0/20:4), were positively associated. Of these, 51 (23 known) lipids remained significant at *Q* <.05.

At follow-up, 185 lipids (53 known) were associated with ABI at *P* <.05. Of the 53 known lipids, 22 lipids, including 13 PCs, 3 TAGs, 2 PEs, FA(15:1), AC(11:1), CER(d33:1), GlcCer(d14:1[4E]/20:0[2OH]), and CE(22:5) B were inversely associated with ABI at *P* <.05, whereas 31 lipids, including 9 PEs, 5 PIs, 4 GlcCers, 3 ACs, 2 CEs, 3 SMs, 2 FAs, and CER(d42:2) B, were positively associated. Of these, 39 (9 known) lipids remained significant at *Q* <.05.

**Figure 4:**
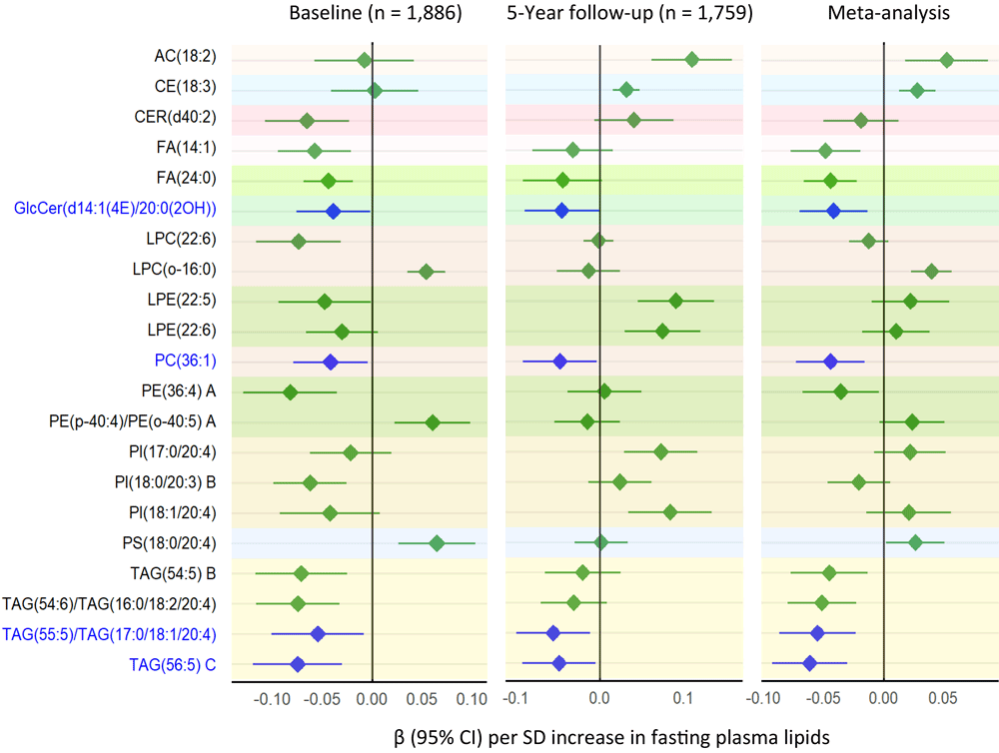
Top-ranked plasma lipids associated with ABI at *P* < .05 identified at baseline or 5-year follow-up. Lipids significantly associated with ABI (*P* < .05) at baseline, at follow-up, and in the meta-analysis are highlighted in blue. “A,” “B,” or “C” in name of lipids indicates isomers. Abbreviations: ABI, ankle-brachial index; AC; acylcarnitine; CE, cholesterol ester; CER, ceramide; eGFR, estimated glomerular filtration rate; FA, fatty acid; GlcCer, glycosylceramide; HR, hazard ratio; LPC, lysophosphatidylcholine; LPE, lysophosphatidylethanolamine; PC, phosphatidylcholine; PE, phosphatidylethanolamine; PI, phosphatidylinositol; SM, sphingomyelin; TAG, triacylglycerol.

Meta-analysis combining results from both time points showed that 14 lipids, including 7 TAGs, 3 FAs, and LPC(p-18:0)/LPC(o-18:1), were inversely associated with ABI, whereas 2 CEs and LPC(o-16:0) were positively associated at *Q* <.05 (Figure 4).

### Results from sensitivity analyses

Additional adjustments for clinical lipids (ie, HDL cholesterol, triglycerides) and physical activity did not attenuate the observed associations. We did not observe significant sex difference in the associations of identified lipids with ABI or change in ABI. In addition, most lipids associated with change in ABI or ABI remained significant even after excluding symptomatic participants or adjusting for symptoms.

## Discussion

In this first large-scale longitudinal lipidomic profiling of subclinical PAD, assessed by change in ABI, among American Indians, we had several significant findings. First, we found that baseline levels of multiple lipid species (eg, glycerophospholipids, glycerolipids, FAs, and SMs) were significantly associated with change in ABI beyond traditional risk factors. Some identified lipids (ie, TAG(48:2), TAG(55:1), PI(16:0/20:4), and cholesterol) were also significantly associated with risk of CHD during an average of 18-years follow-up. Second, our repeated measurement analysis showed, for the first time, that longitudinal changes in several lipid species (ie, ACs, CEs, glycerophospholipids) were significantly associated with change in ABI, independent of clinical factors, baseline ABI, and baseline lipids. Third, cross-sectional analysis showed that altered levels of ACs, FAs, glycerophospholipids (ie, PCs, PEs, PIs), and TAGs were significantly associated with ABI among American Indians. Together, our results could shed light on lipidomic markers associated with subclinical PAD and deepen our understanding of how dyslipidemia may contribute to the development of PAD.

We observed that higher baseline levels of most glycerophospholipids (ie, PCs, PEs, PIs) were positively associated with change in ABI in American Indians. Moreover, longitudinal changes in glycerophospholipids were significantly associated with cardiometabolic traits such as BMI, blood pressure, fasting blood plasma glucose, and insulin resistance. These findings are in line with previous epidemiologic studies demonstrating that some glycerophospholipids, such as PC(32:1), PC(32:3), PC(36:4), and LPC(16:1), were inversely associated with risk of PAD (16) and CHD (17) in non-Hispanic White and Asian people.

Glycerophospholipids, such as PCs, PEs, and PIs, are key components of apolipoprotein B (ApoB)-containing lipoproteins. These lipids are essential in maintaining membrane structure, fluidity, and cell signaling, and they regulate pathways involved in inflammation and oxidative stress (18). Alterations in the levels of PCs, PEs, and PIs can influence the size, density, and atherogenic potential of lipoproteins such as LDL cholesterol and lipoprotein(a) (Lp[a]), contributing to increased CVD risk. Specifically, PCs can be hydrolyzed by lipoprotenin-associated phospholipase A2 (Lp-PLA2), producing lysophosphatidylcholine (LPC) and oxidized FAs (19). LPC is further converted into lysophosphatidic acid (LPA) by autotaxin, which is often elevated in people with high Lp(a) levels (20). Elevated Lp(a) levels are associated with increased LPA levels (21), promoting vascular inflammation and oxidative stress, contributing to atherosclerotic plaques and PAD (20). Disrupted PE metabolism can induce oxidative stress and endothelial dysfunction in arterial walls, exacerbating vascular pathologies (18). Dysregulation of the PI signaling pathway can result in endothelial dysfunction and increased vascular inflammation, both of which are key factors in the development of PAD (22).

Statin therapy, the most widely recognized cholesterol-lowering treatment for managing CVD, including PAD, can influence glycerophospholipid levels. For instance, previous studies (23,24) showed that statins altered key lipid species such as PC(36:4) and PI(18:0/18:2), both of which are involved in lipoprotein metabolism and cardiovascular risk. Notably, the ratio of PI(18:0/18:2) to PC(38:4) explained 58% of the relative CVD risk reduction associated with pravastatin during a 12-month follow-up, independent of change in LDL cholesterol (23). Our study supports this finding, as we observed that higher baseline level of PI(18:0/18:2) was positively associated with change in ABI. This finding aligns with a previous study (25) identifying PI(18:0/18:2) as a predictor of statin response in patients with familial hypercholesterolemia. These results highlight the potential of monitoring specific lipid species, such as PCs and PIs, as biomarkers for statin efficacy in the management of PAD.

Besides glycerophospholipids, we also found that higher baseline levels of certain long- chain glycerolipids (eg, TAGs, DAGs), which have a higher number of carbon atoms and fewer double bonds, were positively associated with change in ABI, suggesting a protective role against subclinical PAD. This aligns with previous research demonstrating that elevated levels of long-chain unsaturated glycerolipids (eg, TAG [48:2], TAG[55:1], DAG[36:1]) were associated with a reduced risk of CHD (17) in multiple populations. Moreover, we observed novel associations, including 2 lipids (ie, TAG[48:2], TAG[55:1]) that demonstrated a protective effect on subclinical PAD and were also associated with a decreased risk of CHD. Conversely, our cross-sectional analysis found that some TAGs with a higher number of carbon atoms and double bonds (eg, TAG[56:5] C, TAG[55:5]/TAG[17:0/18:1/20:4]) showed an inverse association with ABI. This observation is supported by previous studies reporting that 2 TAGs (ie, TAG[56:5] C and TAG[55:5]) were positively associated with the risk of diabetes in Asian (26) and non-Hispanic White people (27). These results suggest that the composition of specific TAG subtypes may have differential associations with PAD. Further investigation is warranted to validate these findings and deepen our understanding of these associations.

Our findings that baseline levels of SMs were inversely associated with change in ABI corroborate previous studies reporting that higher plasma levels of SMs were associated with an increased risk of atherosclerotic plaque (28) and CHD (29). As the predominant sphingolipids in mammalian cell membranes, SMs are crucial for signal transduction, apoptosis, regulation of inflammation, and the oxidative stress response (30). The transformation of sphingomyelin to ceramide in LDL cholesterol by sphingomyelinase, which triggers ceramide aggregation, could represent an early stage in the development of atherosclerosis (31).

Our repeated measurement analysis revealed, for the first time, the association between longitudinal changes in plasma lipidome and change in ABI, independent of clinical covariates, baseline ABI, and baseline lipids. Specifically, changes in ACs, CEs, and glycerophospholipids (eg, PCs, PEs, PIs) were associated with change in ABI as well as changes in cardiovascular risk factors. Besides the contributions of glycerophospholipids to the change in ABI, changes in several lipid classes, such as ACs and CEs, were also associated with changes in multiple cardiometabolic traits. ACs, which are esters formed by carnitine and fatty acids, serve as transporters that move activated long-chain FAs into mitochondria for β-oxidation, an essential process for cellular energy production (32). An excess of ACs may indicate a bottleneck in the β-oxidation pathway, suggesting potential mitochondrial dysfunction that could contribute to the development of PAD (33). Previous studies (34) showed that plasma concentrations of unsaturated cholesterol esters, such as CE(18:2) and CE(17:1), are elevated in patients with intermittent claudication compared with controls. Our study extends these findings by showing that changes in CE(18:3) were positively associated with changes in ABI among participants without intermittent claudication, suggesting its potential role as an early biomarker for subclinical PAD. Interestingly, no such association with CE(18:3) was observed in symptomatic participants in the prior studies (34). CEs serve as a storage form of cholesterol and are the primary neutral lipids found in lipid droplets. Their accumulation in macrophage foam cells, characterized by cholesterol ester-rich lipid droplets, is a key feature of atherosclerosis (35). This differential association across symptomatic and asymptomatic stages suggests the importance of exploring lipid biomarkers like CE(18:3) to enhance early detection and prevention strategies for PAD across its clinical spectrum.

Although younger than the typical screening age for clinical PAD (around 50 years for smokers and 70 years for nonsmokers) (1), the American Indian population has a disproportionate share of PAD, with nearly twice the prevalence compared with the non-Hispanic White population (36). This elevated prevalence is largely attributed to a higher prevalence of cardiovascular risk factors, including high rates of smoking (>40% were current smokers), obesity (55% at baseline, 60% at follow-up), and diabetes (17.9% at baseline, 23.4% at follow-up). These factors accelerate the onset of subclinical PAD, suggesting the need for early screening and prevention strategies in young, high-risk populations, especially in American Indians.

### Strengths and limitations

Our study has several strengths. The major strength is the longitudinal profiling of plasma lipidome in a large, community-based prospective cohort. To our knowledge, our study is the first and by far the largest longitudinal study examining the relationship between change in plasma lipidome and change in ABI across any racial or ethnic group. Second, we used an untargeted lipidomic approach, quantifying more than 1,500 distinct lipid species across 14 known lipid classes in a large prospective cohort of American Indians. While existing biomarkers such as LDL-C, Lp(a), and ApoB are crucial, these markers, combined with other risk factors such as hypertension and tobacco use, still explain only a small proportion of CVD-related outcomes, including PAD, suggesting that additional unmeasured or unknown factors could improve early detection and prevention efforts. Lipidomics can offer a nuanced view by identifying individual lipid species that may not be identified by standard lipid panels. These newly discovered lipids offer supplementary value and could improve risk prediction beyond established risk factors. Third, our statistical models adjusted for a wide range of traditional PAD risk factors, ensuring that the lipids identified in our study are independent of these risk factors. If validated, these newly identified lipid species may serve as novel biomarkers for PAD prediction and risk stratification. Finally, we performed comprehensive statistical analyses, including cross-sectional, prospective, and repeated measurement analyses, allowing us to thoroughly investigate the association between lipid metabolism and ABI in this understudied population.

However, our study has several limitations. First, although our research identified numerous lipid species, many lipids are unknown. More research is needed to further characterize these unknown lipids if they are deemed of interest. Second, although our statistical models adjusted for many traditional risk factors, we cannot entirely rule out potential confounding by unknown or unmeasured factors. Third, our study included only American Indians, a population with high rates of dyslipidemia (78% among adolescents and young adults vs 30% in in the same age groups in the US overall) (37), diabetes (3-fold higher age-adjusted rate than among the non-Hispanic White population) (38), and smoking (>40% of adults vs 27.4% of non-Hispanic White adults) (39). Due to these unique characteristics, our findings may not be generalizable to other racial or ethnic groups with different genetic backgrounds or environmental exposures. Fourth, due to lack of an external cohort with a similar study design and longitudinal lipidomic data, we could not replicate our findings in an independent cohort. However, the large sample size of the study cohort and the identification of multiple lipid species associated with ABI using different statistical models lend credence to our findings. Fifth, while our study identifies novel lipid biomarkers that may enhance early prevention of PAD, the clinical application of these methods is limited by the emerging nature of lipidomics technology. Broader implementation in clinical practice, particularly in resource-limited settings, will require further validation, cost-effectiveness analyses, and technologic advancements. Finally, the observational nature of our study precludes any inference about the causal role of altered lipid metabolism in PAD pathogenesis.

### Conclusion

In this large-scale longitudinal lipidomic analysis, we have, for the first time, reported associations of multiple individual lipid species with subclinical PAD, independent of traditional risk factors. These findings enhance our understanding of the mechanism through which dyslipidemia may contribute to PAD and provide potential novel biomarkers for early prediction and risk stratification in American Indians, an important but traditionally understudied racial minority population.

## References

[1] Gerhard-Herman MD , Gornik HL , Barrett C , Barshes NR , Corriere MA , Drachman DE , . 2016 AHA/ACC guideline on the management of patients with lower extremity peripheral artery disease: a report of the American College of Cardiology/American Heart Association Task Force on Clinical Practice Guidelines. *Circulation.* 2017;135(12):e726–e779. 27840333 10.1161/CIR.0000000000000471 PMC5477786

[2] Allison MA , Armstrong DG , Goodney PP , Hamburg NM , Kirksey L , Lancaster KJ , ; American Heart Association Council on Peripheral Vascular Disease; Council on Hypertension; and Council on Lifestyle and Cardiometabolic Health. Health disparities in peripheral artery disease: a scientific statement from the American Heart Association. *Circulation.* 2023;148(3):286–296. 10.1161/CIR.0000000000001153 37317860 PMC11520198

[3] Kou M , Ding N , Ballew SH , Salameh MJ , Martin SS , Selvin E , . Conventional and novel lipid measures and risk of peripheral artery disease. *Arterioscler Thromb Vasc Biol.* 2021;41(3):1229–1238. 10.1161/ATVBAHA.120.315828 33504178 PMC8188625

[4] Bertrand C , Saulnier PJ , Potier L , Croyal M , Blanchard V , Gand E , ; SURDIAGENE Study Group. Plasma concentrations of lipoproteins and risk of lower-limb peripheral artery disease in people with type 2 diabetes: the SURDIAGENE study. *Diabetologia.* 2021;64(3):668–680. 10.1007/s00125-020-05326-x 33409569

[5] Tikkanen E , Jägerroos V , Holmes MV , Sattar N , Ala-Korpela M , Jousilahti P , . Metabolic biomarker discovery for risk of peripheral artery disease compared with coronary artery disease: lipoprotein and metabolite profiling of 31 657 individuals from 5 prospective cohorts. *J Am Heart Assoc.* 2021;10(23):e021995. 10.1161/JAHA.121.021995 34845932 PMC9075369

[6] Aboyans V , Criqui MH , Abraham P , Allison MA , Creager MA , Diehm C , ; American Heart Association Council on Peripheral Vascular Disease; Council on Epidemiology and Prevention; Council on Clinical Cardiology; Council on Cardiovascular Nursing; Council on Cardiovascular Radiology and Intervention, and Council on Cardiovascular Surgery and Anesthesia. Measurement and interpretation of the ankle-brachial index: a scientific statement from the American Heart Association. *Circulation.* 2012;126(24):2890–2909. 10.1161/CIR.0b013e318276fbcb 23159553

[7] Resnick HE , Foster GL . Prevalence of elevated ankle-brachial index in the United States 1999 to 2002. *Am J Med.* 2005;118(6):676–679. 10.1016/j.amjmed.2004.11.025 15922701

[8] Resnick HE , Lindsay RS , McDermott MM , Devereux RB , Jones KL , Fabsitz RR , . Relationship of high and low ankle brachial index to all-cause and cardiovascular disease mortality: the Strong Heart Study. *Circulation.* 2004;109(6):733–739. 10.1161/01.CIR.0000112642.63927.54 14970108

[9] North KE , Howard BV , Welty TK , Best LG , Lee ET , Yeh JL , . Genetic and environmental contributions to cardiovascular disease risk in American Indians: the Strong Heart Family Study. *Am J Epidemiol.* 2003;157(4):303–314. 10.1093/aje/kwf208 12578801

[10] Rose GA . The diagnosis of ischaemic heart pain and intermittent claudication in field surveys. *Bull World Health Organ.* 1962;27(6):645–658. 13974778 PMC2555832

[11] Lee ET , Welty TK , Fabsitz R , Cowan LD , Le NA , Oopik AJ , . The Strong Heart Study. A study of cardiovascular disease in American Indians: design and methods. *Am J Epidemiol.* 1990;132(6):1141–1155. 10.1093/oxfordjournals.aje.a115757 2260546

[12] Lee ET , Cowan LD , Welty TK , Sievers M , Howard WJ , Oopik A , . All-cause mortality and cardiovascular disease mortality in three American Indian populations, aged 4–74 years, 1984–1988. The Strong Heart Study. *Am J Epidemiol.* 1998;147(11):995–1008. 10.1093/oxfordjournals.aje.a009406 9620042

[13] Miao G , Zhang Y , Huo Z , Zeng W , Zhu J , Umans JG , . Longitudinal plasma lipidome and risk of type 2 diabetes in a large sample of American Indians with normal fasting glucose: the Strong Heart Family Study. *Diabetes Care.* 2021;44(12):2664–2672. 10.2337/dc21-0451 34702783 PMC8669540

[14] Storey JD , Tibshirani R . Statistical significance for genomewide studies. *Proc Natl Acad Sci USA.* 2003;100(16):9440–9445. 10.1073/pnas.1530509100 12883005 PMC170937

[15] Pencina MJ , D’Agostino RB Sr , D’Agostino RB Jr , Vasan RS . Evaluating the added predictive ability of a new marker: from area under the ROC curve to reclassification and beyond. *Stat Med.* 2008;27(2):157–172. 10.1002/sim.2929 17569110

[16] Semporé WY , Chao De La Barca JM , Hersant J , Ouédraogo N , Yaméogo TM , Henni S , . Exercise-induced plasma metabolomic profiles in patients with peripheral arterial disease. *Front Physiol.* 2021;12:758085. 10.3389/fphys.2021.758085 34867463 PMC8637284

[17] Qin M , Zhu Q , Lai W , Ma Q , Liu C , Chen X , . Insights into the prognosis of lipidomic dysregulation for death risk in patients with coronary artery disease. *Clin Transl Med.* 2020;10(5):e189. 10.1002/ctm2.189 32997403 PMC7522592

[18] Calzada E , Onguka O , Claypool SM . Phosphatidylethanolamine metabolism in health and disease. *Int Rev Cell Mol Biol.* 2016;321:29–88. 10.1016/bs.ircmb.2015.10.001 26811286 PMC4778737

[19] Norris PC , Gosselin D , Reichart D , Glass CK , Dennis EA . Phospholipase A2 regulates eicosanoid class switching during inflammasome activation. *Proc Natl Acad Sci USA.* 2014;111(35):12746–12751. 10.1073/pnas.1404372111 25139986 PMC4156727

[20] Huang F , Wang K , Shen J . Lipoprotein-associated phospholipase A2: the story continues. *Med Res Rev.* 2020;40(1):79–134. 10.1002/med.21597 31140638 PMC6973114

[21] Dzobo KE , Cupido AJ , Mol BM , Stiekema LCA , Versloot M , Winkelmeijer M , . Diacylglycerols and lysophosphatidic acid, enriched on lipoprotein(a), contribute to monocyte inflammation. *Arterioscler Thromb Vasc Biol.* 2024;44(3):720–740. 10.1161/ATVBAHA.123.319937 38269588 PMC10880937

[22] Annex BH , Cooke JP . New directions in therapeutic angiogenesis and arteriogenesis in peripheral arterial disease. *Circ Res.* 2021;128(12):1944–1957. 10.1161/CIRCRESAHA.121.318266 34110899 PMC8538391

[23] Jayawardana KS , Mundra PA , Giles C , Barlow CK , Nestel PJ , Barnes EH , ; LIPID Study Investigators. Changes in plasma lipids predict pravastatin efficacy in secondary prevention. *JCI Insight.* 2019;4(13):e128438. 10.1172/jci.insight.128438 31292301 PMC6629250

[24] Schooneveldt YL , Giles C , Keating MF , Mellett NA , Jurrjens AW , Paul S , . The impact of simvastatin on lipidomic markers of cardiovascular risk in human liver cells is secondary to the modulation of intracellular cholesterol. *Metabolites.* 2021;11(6):340. 10.3390/metabo11060340 34070445 PMC8228384

[25] Cerda A , Bortolin RH , Yoshinaga MY , Freitas RCC , Dagli-Hernandez C , Borges JB , . Lipidomic analysis identified potential predictive biomarkers of statin response in subjects with familial hypercholesterolemia. *Chem Phys Lipids.* 2023;257:105348. 10.1016/j.chemphyslip.2023.105348 37827478

[26] Lu J , Lam SM , Wan Q , Shi L , Huo Y , Chen L , . High-coverage targeted lipidomics reveals novel serum lipid predictors and lipid pathway dysregulation antecedent to type 2 diabetes onset in normoglycemic Chinese adults. *Diabetes Care.* 2019;42(11):2117–2126. 10.2337/dc19-0100 31455687

[27] Fernandez C , Surma MA , Klose C , Gerl MJ , Ottosson F , Ericson U , . Plasma lipidome and prediction of type 2 diabetes in the population-based Malmö Diet and Cancer cohort. *Diabetes Care.* 2020;43(2):366–373. 10.2337/dc19-1199 31818810

[28] Edsfeldt A , Dunér P , Ståhlman M , Mollet IG , Asciutto G , Grufman H , . Sphingolipids contribute to human atherosclerotic plaque inflammation. *Arterioscler Thromb Vasc Biol.* 2016;36(6):1132–1140. 10.1161/ATVBAHA.116.305675 27055903

[29] Jiang XC , Paultre F , Pearson TA , Reed RG , Francis CK , Lin M , . Plasma sphingomyelin level as a risk factor for coronary artery disease. *Arterioscler Thromb Vasc Biol.* 2000;20(12):2614–2618. 10.1161/01.ATV.20.12.2614 11116061

[30] Slotte JP . Biological functions of sphingomyelins. *Prog Lipid Res.* 2013;52(4):424–437. 10.1016/j.plipres.2013.05.001 23684760

[31] Devlin CM , Leventhal AR , Kuriakose G , Schuchman EH , Williams KJ , Tabas I . Acid sphingomyelinase promotes lipoprotein retention within early atheromata and accelerates lesion progression. *Arterioscler Thromb Vasc Biol.* 2008;28(10):1723–1730. 10.1161/ATVBAHA.108.173344 18669882 PMC2562252

[32] Meierhofer D . Acylcarnitine profiling by low-resolution LC-MS. *PLoS One.* 2019;14(8):e0221342. 10.1371/journal.pone.0221342 31415665 PMC6695155

[33] Bonaca MP , Hamburg NM , Creager MA . Contemporary medical management of peripheral artery disease. *Circ Res.* 2021;128(12):1868–1884. 10.1161/CIRCRESAHA.121.318258 34110910

[34] Ismaeel A , Franco ME , Lavado R , Papoutsi E , Casale GP , Fuglestad M , . Altered metabolomic profile in patients with peripheral artery disease. *J Clin Med.* 2019;8(9):1463. 10.3390/jcm8091463 31540015 PMC6780416

[35] Zadoorian A , Du X , Yang H . Lipid droplet biogenesis and functions in health and disease. *Nat Rev Endocrinol.* 2023;19(8):443–459. 10.1038/s41574-023-00845-0 37221402 PMC10204695

[36] Hackler EL III , Hamburg NM , White Solaru KT . Racial and ethnic disparities in peripheral artery disease. *Circ Res.* 2021;128(12):1913–1926. 10.1161/CIRCRESAHA.121.318243 34110901

[37] Reese JA , Roman MJ , Deen JF , Ali T , Cole SA , Devereux RB , . Dyslipidemia in American Indian Adolescents and Young Adults: Strong Heart Family Study. *J Am Heart Assoc.* 2024;13(6):e031741. 10.1161/JAHA.123.031741 38445515 PMC11010025

[38] Breathett K , Sims M , Gross M , Jackson EA , Jones EJ , Navas-Acien A , ; American Heart Association Council on Epidemiology and Prevention; Council on Quality of Care and Outcomes Research; Council on Cardiovascular and Stroke Nursing; Council on Clinical Cardiology; and Council on Lifestyle and Cardiometabolic Health. Cardiovascular health in American Indians and Alaska Natives: a scientific statement from the American Heart Association. *Circulation.* 2020;141(25):e948–e959. 10.1161/CIR.0000000000000773 32460555 PMC7351358

[39] Jernigan VB , Duran B , Ahn D , Winkleby M . Changing patterns in health behaviors and risk factors related to cardiovascular disease among American Indians and Alaska Natives. *Am J Public Health.* 2010;100(4):677–683. 10.2105/AJPH.2009.164285 20220114 PMC2836357

